# Assessing the Vaccination Status and Barriers to Influenza, Pneumococcal, and COVID-19 Vaccination Among Moroccan Patients With Chronic Inflammatory Rheumatic Disease

**DOI:** 10.7759/cureus.61676

**Published:** 2024-06-04

**Authors:** Samya Ez-zaoui, Hanan Rkain, Fatine Kronbi, Nada Benzine, Sara Farih, Latifa Tahiri, Redouane Abouqal, Kenza Hassouni, Najia Hajjaj-Hassouni, Fadoua Allali

**Affiliations:** 1 Rheumatology, Ayachi Hospital, Ibn Sina Hospital Center, Rabat, MAR; 2 Rheumatology, Faculty of Medicine and Pharmacy, Mohammed V University, Rabat, MAR; 3 Exercise Physiology and Autonomous Nervous System, Faculty of Medicine and Pharmacy, Mohammed V University, Rabat, MAR; 4 Biostatistics, Clinical and Epidemiological Research, Faculty of Medicine and Pharmacy, Mohammed V University, Rabat, MAR; 5 Social Sciences, International School of Public Health, Mohammed VI University of Health Sciences, Casablanca, MAR; 6 Rheumatology, Faculty of Medicine, Research Center of Health Sciences, International University of Rabat, Rabat, MAR

**Keywords:** reasons for non-vaccination, chronic inflammatory rheumatic disease, covid-19 vaccine, pneumococcal vaccine, influenza vaccine

## Abstract

Objective: To evaluate the vaccination coverage of patients with chronic inflammatory rheumatic disease (CIRD) against influenza, pneumococcus, and COVID-19 and to determine, per the patients' point of view, the possible factors related to vaccination hesitation and/or refusal.

Methods: A cross-sectional study carried out by the vaccination working group of the Moroccan Society of Rheumatology, including patients with CIRD in Morocco. Information about vaccination coverage and reasons for non-vaccination against influenza, pneumococcal infection, and COVID-19 was collected.

Results: This survey included 230 patients (mean age of 46.9 +/-13.89 years; 68.7% females) affected by CIRD (rheumatoid arthritis 53%, spondyloarthritis 39.6%, psoriatic arthritis 7%). The study shows a significant lack of influenza and pneumococcal vaccination in CIRD patients, with vaccination coverage against influenza, pneumococcal infection, and COVID-19 at 2.2%, 0.4%, and 80.9%, respectively. The main reason for non-vaccination against influenza and pneumococcus was related to the absence of recommendations by their doctors (77%, 87%, p = 0.04). Additionally, the primary reason for non-vaccination against COVID-19 was the fear of the vaccine's side effects (51%, p = 0.0001), mainly a flare-up of CIRD (44%, p = 0.001).

Conclusion: This survey shows a lack of influenza, pneumococcal, and COVID-19 vaccination in CIRD patients. The principal actions to improve vaccination should aim to educate patients and encourage rheumatologists to vaccinate their patients.

## Introduction

Patients with chronic inflammatory rheumatic diseases (CIRDs) have a significantly increased risk of infectious complications, posing a major concern for morbidity and mortality within this population [1]. This heightened susceptibility to infections stems from a combination of associated comorbidities and the use of immunosuppressive therapies to manage chronic inflammation [2].

Epidemiological data from a US study highlights that patients with CIRDs have an elevated risk of contracting influenza, estimated to be approximately 20% higher than the general population [3]. Additionally, the risk of developing pneumococcal pneumonia is markedly increased, up to three to four times compared to those without CIRDs [4]. Moreover, a recent publication has demonstrated that patients with autoimmune diseases are at an elevated risk of experiencing severe COVID-19 complications, despite their risk of contracting the infection being comparable to that of healthy individuals who are 20 years older. This finding underscores the importance of increased vigilance and strengthened preventive measures among autoimmune disease patients to mitigate the risk of developing severe COVID-19 outcomes [5].

In the face of these heightened infectious risks, vaccination represents a critical prevention strategy. It plays a pivotal role in the overall management of patients with CIRDs, albeit vaccination coverage often being suboptimal [6,7]. It is recommended that patients with CIRDs receive, in addition to routine vaccinations, three specific vaccinations, i.e., against seasonal influenza annually, against pneumococcus (with a simplified vaccination schedule due to new recommendations regarding the 13-valent pneumococcal conjugate vaccine (PCV)13 conjugate vaccine in adults), and against COVID-19 [1-2].

Despite clear evidence of vaccine efficacy and safety, vaccination coverage, particularly against seasonal influenza, remains well below the 75% target set by the WHO for this population [8]. Therefore, it is crucial to understand barriers to vaccination to design interventions aimed at improving vaccination coverage among patients with CIRDs. This study specifically aims to evaluate vaccination coverage among patients with CIRDs against influenza, pneumococcus, and COVID-19. Additionally, it seeks to identify and understand the underlying reasons for non-vaccination, based on patient perspectives. By exploring these factors, we can develop more targeted strategies to overcome vaccination barriers and enhance protection against infections for patients with CIRDs.

## Materials and methods

This is a cross-sectional, descriptive, and analytical study, including 230 patients with CIRD. Data regarding the characteristics of patients and their CIRDs, the rate of vaccination against seasonal influenza, pneumococcus, and COVID-19, and reasons for non-vaccination from the patient's perspective was collected using a questionnaire. This study was conducted by the Rheumatology B team at Ayachi Hospital and the Moroccan Association for Research and Assistance to Rheumatic Patients (AMRAR), with the vaccination working group of the Moroccan Society of Rheumatology. We conducted a telephone survey to reach patients with chronic inflammatory rheumatic diseases. The inclusion criteria were patients with chronic inflammatory rheumatism aged over 18 years of age. The exclusion criteria were the refusal of patients with CIRDs to participate in the study.

The survey received approval from the Ethics Committee of the Faculty of Medicine and Pharmacy, Mohammed V University, Rabat, Morocco (approval no. D/24) and was conducted per the ethical standards of the 1964 Declaration of Helsinki and its later amendments or comparable standards. Each patient received an information letter and consent form detailing the purpose and process of the study, along with a clickable link to the survey. Completing the self-administered questionnaire implied consent to use the responses, and all data were analyzed anonymously.

Questionnaire

We designed a questionnaire for patients with CIRDs containing closed-ended questions distributed across four sections. The first section collected sociodemographic characteristics and patient history: age, gender, level of education (illiterate, primary, secondary, university), habitat (rural, urban), social coverage, comorbidities, and history of severe pulmonary infection requiring hospitalization or intravenous treatment. The second section gathered disease characteristics, namely the type of CIRD), duration of illness, duration of follow-up by a rheumatologist, regularity of follow-up, and current treatments. The third section addressed the vaccination schedule of patients with CIRDs: vaccination against seasonal influenza during the winter (2022-2023), history of influenza vaccination in previous winters, pneumococcal vaccination in the past five years, COVID-19 vaccination, including the number of doses received, and whether the rheumatologist and/or general practitioner recommended or prescribed the vaccine. The fourth and final section explored the various reasons preventing patients with CIRDs from being vaccinated against seasonal influenza, pneumococcus, and COVID-19, including fear of vaccine side effects, fear of CIRD flare-ups post-vaccination, forgetfulness, reluctance to get vaccinated, patient attitudes towards vaccination, lack of means to get vaccinated, and non-recommendation by the treating physician.

We opted for an electronic questionnaire format to facilitate its dissemination. Thus, the questionnaire was drafted on Google Forms (Google LLC, Mountain View, CA, USA) platform with a direct link for access. This link was distributed to patients through the AMRAR associations, telephone contacts, and social networks (WhatsApp and Facebook). When inviting patients to participate in our study, we provided them with information about the objective of our work, the simplicity of completing the questionnaire, and the voluntary, anonymous, and confidential nature of our survey. Patient participation was conditional upon approved consent. Patients were free to refuse to participate and/or leave the survey at any time. The questionnaire is available in Appendix A.

Statistics

The data from all questionnaire responses were entered into Microsoft Excel (Microsoft Corp., Redmond, WA, USA) and analyzed using the statistical software SPSS Statistics version 20 (IBM Corp., Armonk, NY, USA). We conducted descriptive and analytical statistical analyses. Quantitative variables were expressed as means ± standard deviation (SD) or medians depending on their Gaussian or non-Gaussian distribution. Qualitative variables were expressed as percentages. A comparison of qualitative variables was performed using the chi-square test. The comparison of quantitative variables was conducted using correlation analysis with Pearson's parametric test for variables with a Gaussian distribution and Spearman's non-parametric test for those with a non-Gaussian distribution. A comparison was also made between quantitative and qualitative variables according to their distribution using the Student's t-test or the Mann-Whitney non-parametric test. A difference was considered statistically significant at p ≤ 0.05.

## Results

Characteristics of CIRD patients

The sociodemographic characteristics of the population and their comorbidities are depicted in Table 1. The mean age of patients was 46.9 ± 13.89 years, with females representing 68.7% of the study population. The illiteracy rate was 27%. Comorbidities found in our study population, in descending order, included diabetes (14.8%), cardiovascular diseases (11.3%), allergies (13%), asthma (5.7%), and a history of severe pulmonary infection in 4.8% of patients.

**Table 1:** Characteristics of rheumatic patients *Expressed as mean and standard deviation

Patient characteristics and comorbidities	N = 230
Age (years) *	46.9 +/-13.89
Female sex (%)	68.7
Education level	
Illiterate (%)	27
Primary (%)	19.1
Secondary (%)	26.9
University (%)	27
Urban area (%)	27
Comorbidities	
Diabetes (%)	14.8
Hypertension (%)	11.3
Asthma (%)	5.7
History of pulmonary infection requiring hospitalization (%)	4.8
Allergy (%)	13
Nephropathy (%)	2.6
Hepatopathy (%)	1.3

Characteristics of CIRD

Among the 230 included patients, 53.4% were being treated for rheumatoid arthritis (RA), 39.6% for axial spondyloarthritis (AxSpA), and 7% for psoriatic arthritis. The response rate to the study questionnaire was 100%, with no patient refusing to respond. The mean duration of their diseases was 11.9 ± 7.9 years, with 86% receiving regular follow-up with a rheumatologist.

Regarding current treatments, 35% of patients were on non-steroidal anti-inflammatory drugs, 60.4% were on corticosteroid therapy with a median dose of 2 mg/day, and 15% were taking more than 10 mg/day. Concerning conventional treatments, 45.2% of patients were on methotrexate, 17% on sulfasalazine, 8.7% on leflunomide, and 0.4% on hydroxychloroquine. Regarding biologic treatments, 47% of the included patients were on biologic therapy, including 7.4% on rituximab, 10.4% and 20.9% on anti-tumor necrosis factor (TNF)-alpha (intravenous and subcutaneous, respectively), 4.3% on anti-IL17, 4.8% on anti-IL6, and 0.9% on anti-IL1.

Vaccination schedule of CIRD patients

This survey shows that only 2.2% of patients had received the seasonal influenza vaccine during the winter of 2022-2023, compared to 0.4% of patients who received the pneumococcal vaccine in the past five years, equivalent to one patient out of 230. Furthermore, only 14.8% and 0.4% of patients were advised by their treating rheumatologists to be vaccinated against seasonal influenza and pneumococcus, respectively. Additionally, 6.5% and 0.4% of patients received a prescription for the influenza and pneumococcal vaccines, respectively, from their treating physicians.

**Figure 1:**
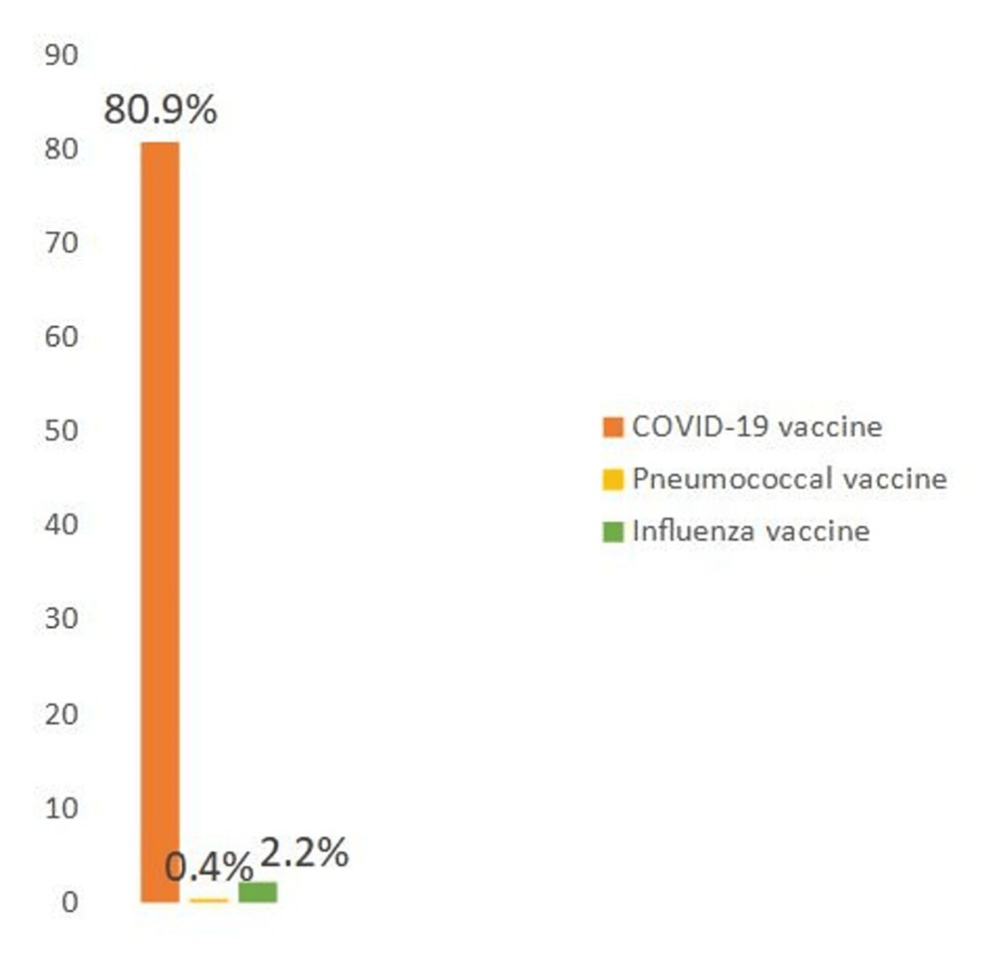
Vaccination coverage rates against influenza, pneumococcus, and COVID-19

Regarding the COVID-19 vaccination rate, 80.9% of included patients received the COVID-19 vaccine, with 2.6%, 44.8%, 32.6%, and 1.3% receiving one, two, three, and four doses, respectively. More than half of the patients (58.7%) were advised by their treating rheumatologists to be vaccinated against COVID-19. Figure 1 illustrates the vaccination coverage rates against influenza, pneumococcus, and COVID-19.

Reasons for non-vaccination among CIRD patients

The primary reason for non-vaccination against influenza and pneumococcus was related to the non-recommendation by physicians, including the absence of vaccine advice and prescription (77%, 86.1%), with a significant p-value of 0.004. Conversely, the main reason for non-vaccination against COVID-19 was fear of vaccine side effects (51%, p = 0.0001), particularly fear of CIRD flare-ups (63.9%, p = 0.001).

**Figure 2:**
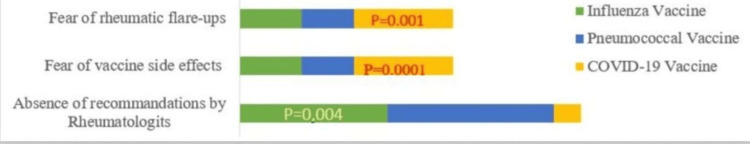
Factors associated with vaccination hesitation and/or the refusal to get vaccinated

The remaining reasons cited by patients for not getting vaccinated against seasonal influenza, pneumococcus, and COVID-19 included refusal of the vaccination concept (14.3%, 11.3%, 24.8%, respectively), lack of interest in vaccination (29.1%, 21.7%, 30.9%, respectively), forgetting to get vaccinated (5.7%, 3.9%, 2.2%, respectively), laziness to get vaccinated (9.1%, 6.1%, 8.3%, respectively), and lack of means (8.3%, 8.7%, respectively). Figure 2 illustrates the main reasons for non-vaccination against influenza, pneumococcus, and COVID-19 from the patients' perspectives.

## Discussion

Our study provides a comprehensive overview of vaccination coverage against influenza, pneumococcus, and COVID-19 among Moroccan patients with CIRDs. It also identifies the main barriers to non-vaccination from the patient's perspective.

Patients with CIRDs experience immune system dysregulation, leading to increased susceptibility to infections, a phenomenon closely linked to comorbidities [6,7] and immunosuppressive therapy, including glucocorticoids (GC), conventional synthetic disease-modifying antirheumatic drugs (csDMARDs), and biologic (bDMARDs) and targeted therapies (targeted synthetic (ts)DMARDs). Therefore, infection prevention is considered a crucial issue in managing patients with CIRDs [6-9]. Vaccination remains a cornerstone in preventing infections [10,11], and plays a significant role in the management of CIRD patients, who universally exhibit suboptimal vaccine adoption rates [12].

Our study reveals a very low rate of influenza (2.4%) and pneumococcal (0.4%) vaccination coverage among Morrocan CIRD patients, far below international guidelines [2-7] regarding vaccination in rheumatic patients. These findings echo those of the international COMORA cohort published in 2015, which showed low optimal vaccination rates for both pneumococcal and influenza vaccines, with substantial disparities between countries [9]. For instance, vaccination rates ranged from 0% in Morocco to 56.5% in France for pneumococcal vaccination and from less than 1% in Morocco and Egypt to 66.2% in Japan for influenza vaccination [9]. Additionally, a French study published in 2015, involving 584 patients on biologic therapy, reported influenza and pneumococcal vaccination coverage rates of 44% and 62%, respectively [13]. Furthermore, several other studies have highlighted inadequate vaccination rates against influenza and pneumococcus, with rates ranging from 28% to 78% for influenza vaccination [14,15], and 9% to 78% for pneumococcal vaccination [16].

However, the COVID-19 vaccination rate was satisfactory in our study, with a vaccination rate of 89%, consistent with the results of several recent studies following the national COVID-19 vaccination campaign. A multicenter study published in 2021 reported a COVID-19 vaccination coverage rate of 87% [17]. Another study published in 2022 concluded a COVID-19 vaccination coverage rate of 80% among patients with CIRDs [17]. Similarly, a study conducted in Germany in 2022 found a COVID-19 vaccination coverage rate of 85% among patients with CIRDs [18]. This satisfactory COVID-19 vaccination rate underscores the importance of awareness campaigns during the COVID-19 pandemic in Morocco [8].

Our study also aimed to identify barriers to non-vaccination against influenza, pneumococcus, and COVID-19. We found that the primary reason for non-vaccination against influenza and pneumococcus was significantly related to the lack of recommendations by physicians, including the absence of vaccine advice and prescriptions. In contrast, the main reason for non-vaccination against COVID-19 was fear of vaccine side effects, particularly concerns about CIRD flare-ups. Other reasons cited by patients included refusal of vaccination, lack of interest, forgetfulness, laziness, and financial constraints. Our results are consistent with previous studies. For example, a study published in 2021 on 166 CIRD patients found that the primary reason for non-vaccination against influenza and pneumococcus was the lack of vaccine recommendation by the treating physician, followed by fear of side effects and vaccine refusal. Regarding COVID-19 vaccination, fear of the new vaccine and lack of recommendation by the treating physician were the main reasons for non-vaccination [15]. These findings align with those of a Tunisian study published in 2018, which identified lack of information provided by the treating physician and fear of side effects as the main reasons preventing patients from being vaccinated [14]. Similarly, a French study published in 2015 on vaccination coverage among CIRD patients receiving biologic therapy reported that forgetfulness and fear of side effects were the main reasons for non-vaccination against influenza and pneumococcus [13].

Our study has several limitations that need to be acknowledged. First, being a cross-sectional study, it only provides a snapshot of vaccination coverage at a specific point in time, and causality cannot be inferred. Additionally, the study relied on self-reported data, which may introduce recall bias or social desirability bias, particularly regarding vaccination status and reasons for non-vaccination. Moreover, the study was conducted at a single center, which may limit the generalizability of the findings to other settings or populations. We acknowledge that the use of social networks in our study can introduce limitations, such as recall bias. However, we would like to clarify that we have implemented robust measures to ensure participant anonymity.

Despite these limitations, our study investigated vaccination coverage and barriers among patients with CIRDs, revealing critical insights into preventive healthcare in this population. By comprehensively assessing vaccination against influenza, pneumococcus, and COVID-19, our study provides a holistic understanding of vaccination patterns among CIRD patients, contributing significantly to the existing literature.

The low rates of influenza and pneumococcal vaccination underscore substantial gaps in adherence to international vaccination guidelines among CIRD patients. These findings emphasize the urgent need for targeted interventions to improve vaccination uptake and mitigate the risk of infectious complications in this vulnerable population. Furthermore, the satisfactory COVID-19 vaccination coverage highlights the success of awareness campaigns during the pandemic and underscores the importance of proactive vaccination efforts.

Identifying physician recommendation as a key determinant of vaccination behavior highlights the pivotal role of healthcare providers in promoting preventive healthcare among CIRD patients. Addressing patient concerns, such as fear of vaccine side effects and disease flare-ups, is crucial for enhancing vaccine acceptance and adherence.

Understanding factors hindering vaccination of patients with CIRDs may help in developing targeted interventions. For example, based on our study, we could suggest that we first need to raise awareness among rheumatologists and then among patients to ensure proper vaccination, while working on the misconceptions circulating or sometimes mediatized (such as negative stories reported in the media that have had a strong influence on the decision to be vaccinated), improving patients' knowledge of the various vaccines and their interest in preventing infections, and scheduling special vaccination consultations [19].

## Conclusions

Our study underscores the critical importance of vaccination in mitigating infectious complications among patients with CIRDs. Despite international recommendations, our findings reveal a concerning lack of influenza and pneumococcal vaccination uptake in this vulnerable population of Moroccan patients. This deficiency is particularly alarming given the heightened susceptibility of CIRD patients to infectious morbidity and mortality. To address this issue, targeted interventions are imperative. It is essential to prioritize the education of both rheumatologists and patients, emphasizing the significance of vaccination in preventing infectious diseases and dispelling prevalent misconceptions. Additionally, dedicated vaccination consultations may enhance vaccine uptake by providing tailored guidance and addressing patient concerns. Ultimately, by fostering physician engagement and implementing targeted educational initiatives, we can improve vaccination rates among CIRD patients, thereby reducing the burden of infectious diseases and enhancing overall healthcare outcomes in this population.

## Appendices

Appendix A

Below is the questionnaire for patients with CIRDs containing closed-ended questions distributed across four sections.

Patient characteristics

Age (Years):

Sex: 

o   Female    

o   Male

Place of residence:

o   Urban

o   Rural

What is your level of education?

o   Primary

o   Secondary

o    University 

o    Illiterate

Personal history:

Hypertension:

o   Yes

o   No

Diabetes:

o   Yes

o   No

Heart disease:

o   Yes

o   No

History of serious pulmonary infection requiring hospitalization or venous treatment:

o   Yes

o   No

Asthma:

o   Yes

o   No

Allergy:

o   Yes

o   No

Kidney disease:

o   Yes

o   No

Hepatopathy:

o   Yes

o   No

Chronic inflammatory rheumatic disease characteristics

Which chronic inflammatory rheumatism are you being treated for?

Rheumatoid arthritis:

o   Yes

o   No

Spondyloarthropathy:

o   Yes

o   No

Psoriatic arthritis:

o   Yes

o   No

Length of time your rheumatism has progressed (in years):

Length of time your rheumatism has been followed up by a rheumatologist (in years):

Medical follow-up conducted by your rheumatologist:

o   Regular

o   Non-regular

Current treatment:

Non-steroidal anti-inflammatory drugs:

o   Yes

o   No

Corticosteroid therapy:

o   Yes

o   No

If yes, what is your current dose of corticosteroid therapy (in mg)?

Conventional Synthetic Disease-Modifying Antirheumatic Drugs (csDMARDs)

Methotrexate :

o   Yes

o   No

Arava:

o   Yes

o   No

Salazopyrine:

o   Yes

o   No

Plaquenil:

o   Yes

o   No

Biologic  therapies (bDMARDs)

o   Yes

o   No

If yes, which?

Rituximab:

o   Yes

o   No

Intravenous anti-TNF alpha:

o   Yes

o   No

Subcutaneous anti-TNF alpha:

o   Yes

o   No  

Anti IL17:

o   Yes

o   No

Anti IL6:

o   Yes

o   No

Anti IL1:

o   Yes

o   No

Anti JAK:

o   Yes

o   No

Vaccination schedule for patients with chronic inflammatory rheumatism disease

Influenza Vaccine

Have you received the seasonal influenza vaccine for this winter (2022-2023)?

o   Yes

o   No

Have you already received the influenza flu vaccine for previous winters during the course of your rheumatism?

o   Yes

o   No

Has your doctor ever advised you to be vaccinated against seasonal influenza?

o   Yes

o   No

If yes, is it your:

o   General practitioner

o   Rheumatologist

Has your doctor already prescribed the flu vaccine?

o   Yes

o   No

If yes, is it your

o   General practitioner

o   Rheumatologist

Pneumococcal Vaccine

Have you received the pneumococcal vaccine in the last five years?

o   Yes

o   No

Has your doctor ever advised you to be vaccinated against pneumococcus?

o   Yes

o   No

If yes, was it your:

o   General practitioner

o   Rheumatologist

Has your doctor already prescribed the pneumococcal vaccine?

o   Yes

o   No

If yes, is it your:

o   General practitioner

o   Rheumatologist

COVID-19 Vaccine

Have you received the COVID-19 vaccine?

o   Yes

o   No

If yes, how many doses?

o   1

o   2

o   3

o   4

Has your doctor advised you to be vaccinated against COVID-19?

o   Yes

o   No

If yes, was it your:

o   General practitioner

o   Rheumatologist

Reasons for non-vaccination against influenza

My doctor has never advised me to get vaccinated against seasonal influenza

o   Yes

o   No

My doctor has never prescribed the influenza vaccine

o   Yes

o   No

I'm afraid of the side effects of the influenza vaccine

o   Yes

o   No

I'm afraid that the influenza vaccine will revive my rheumatism

o   Yes

o   No

I don't really see the point of seasonal influenza vaccination

o   Yes

o   No

I'm against influenza vaccination

o   Yes

o   No

I forget to get vaccinated against influenza

o   Yes

o   No

I'm too lazy to get an influenza shot

o   Yes

o   No

I don't have enough money to get an influenza shot 

o   Yes

o   No

Reasons for non-vaccination against pneumococcus

My doctor has never advised me to be vaccinated against pneumococcus

o   Yes

o   No

My doctor never prescribed the pneumococcal vaccine

o   Yes

o   No

I'm afraid of the side effects of the pneumococcal vaccine

o   Yes

o   No

I'm afraid that the pneumococcal vaccine will revive my rheumatism

o   Yes

o   No

I don't really see the point of pneumococcal vaccination

o   Yes

o   No

I'm against pneumococcal vaccination

o   Yes

o   No

I forget to get vaccinated against pneumococcus

o   Yes

o   No

I'm too lazy to get vaccinated against pneumococcus

o   Yes

o   No

I don't have enough money to get vaccinated against pneumococcus

o   Yes

o   No

Reasons for non-vaccination against COVID-19

My doctor has never advised me to be vaccinated against COVID-19

o   Yes

o   No

My doctor never prescribed the COVID-19 vaccine

o   Yes

o   No

I am afraid of the side effects of the COVID-19 vaccine

o   Yes

o   No

I'm afraid the COVID-19 vaccine will revive my rheumatism

o   Yes

o   No

I don't really see the point of the COVID-19 vaccination

o   Yes

o   No

I am against COVID-19 vaccination

o   Yes

o   No

I forget to get vaccinated against COVID-19

o   Yes

o   No

I'm too lazy to get vaccinated against COVID-19

o   Yes

o   No
